# Magnetic Resonance Imaging Detects Placental Hypoxia and Acidosis in Mouse Models of Perturbed Pregnancies

**DOI:** 10.1371/journal.pone.0059971

**Published:** 2013-03-26

**Authors:** Gabriele Bobek, Tim Stait-Gardner, Laura Surmon, Angela Makris, Joanne M. Lind, William S. Price, Annemarie Hennessy

**Affiliations:** 1 School of Medicine, University of Western Sydney, Campbelltown, New South Wales, Australia; 2 Nanoscale Organisation and Dynamics, School of Science and Health, University of Western Sydney, Penrith, New South Wales, Australia; 3 The Heart Research Institute, University of Sydney, Sydney, New South Wales, Australia; 4 Liverpool Hospital, Renal Unit Liverpool, Liverpool, New South Wales, Australia; Otto-von-Guericke University Magdeburg, Germany

## Abstract

Endothelial dysfunction as a result of dysregulation of anti-angiogenic molecules secreted by the placenta leads to the maternal hypertensive response characteristic of the pregnancy complication of preeclampsia. Structural abnormalities in the placenta have been proposed to result in altered placental perfusion, placental oxidative stress, cellular damage and inflammation and the release of anti-angiogenic compounds into the maternal circulation. The exact link between these factors is unclear. Here we show, using Magnetic Resonance Imaging as a tool to examine placental changes in mouse models of perturbed pregnancies, that *T*_2_ contrast between distinct regions of the placenta is abolished at complete loss of blood flow. Alterations in *T*_2_ (spin-spin or transverse) relaxation times are explained as a consequence of hypoxia and acidosis within the tissue. Similar changes are observed in perturbed pregnancies, indicating that acidosis as well as hypoxia may be a feature of pregnancy complications such as preeclampsia and may play a prominent role in the signalling pathways that lead to the increased secretion of anti-angiogenic compounds.

## Introduction

The placenta is central in the aetiology of preeclampsia, a complication of pregnancy characterised by gestational hypertension and proteinuria and a leading cause of morbidity and mortality in both mothers and infants. It has been postulated that reduced placental perfusion as a result of abnormal placental implantation is the initiating event that leads to the maternal syndrome [1]. More recently, endothelial dysfunction has emerged as the proximal cause for the maternal clinical symptoms, with increased levels of soluble fms-like tyrosine kinase-1 (sFlt-1) secreted by the placenta interfering with the bioavailability of vascular endothelial growth factor (VEGF) and VEGF signalling across the maternal endothelium leading to the maternal hypertensive response [2]. The question however remains concerning the cause of the dysregulation of sFlt-1. Although placental ischaemia and oxidative stress have been shown to have a significant role it remains unclear whether this is an outcome or a cause of structural changes in the placenta and how this links with release of the anti-angiogenic molecules.

Placental vascular visualisation has been difficult and limited to mainly ultrasound Doppler in live animals or recently to *in vivo* multiphoton microscopy [3]. Magnetic Resonance Imaging (MRI) studies of placental anatomy and perfusion have been conducted in humans [4]–[6], mice [7]–[9] and rats [10]. While dynamic contrast-enhanced MRI, using injected contrast agents, has yielded estimates of mean blood flow in the placenta of animals [11] non-invasive techniques such as arterial spin labelling, diffusion imaging and measurements of *T*_1_ (spin-lattice or longitudinal) and *T*_2_ (spin-spin or transverse) relaxation times have been investigated to provide alternative safe techniques for assessing human placental structure and function [12].

A major source of image contrast in MRI studies performed without the use of contrast agents comes from the inherent variation in relaxation times between tissues, as well as contributions from proton density, diffusion and flow. Relaxation results from local fluctuations in magnetic fields, which in turn result from predominantly reorientational and to a lesser extent translational motion of the species containing the nuclear spins. *T*_1_ and *T*_2_ relaxation times are tissue specific with *T*_1_ relaxation times tending to be longest when the protons are “bound” to macromolecules, shorter when they are “free” in solution and shortest when they are in intermediate “structured” states. *T*_2_ relaxation times tend to be longest where protons are “free” in solution and shortest when “bound” to macromolecules [13]. The presence of paramagnetic ions, (e.g., deoxyhaemoglobin), give rise to short *T*_2_ relaxation times [14]–[15] while lower pH gives longer *T*_2_ relaxation times [16]–[17]. Rapidly flowing protons (e.g., flow of arterial blood) will result in loss of signal as the protons move out of the field of view.

In previous studies of *T*_1_ and *T*_2_ relaxation times, human placenta has been reported as appearing homogeneous, with no internal morphology apparent [18]–[20]. These studies involved field strengths of 0.5 or 1.5 Tesla and have shown a correlation of *T*_1_ and *T*_2_ relaxation times with gestational age and a trend for shorter *T*_1_ and *T*_2_ times in pregnancies complicated by preeclampsia and fetal growth restriction [19].

In this study we have used much higher field strength (11.74 Tesla) to investigate whether structural heterogeneities in the placenta could be discerned by *T*_2_ mapping and to determine if perfusion is a determinant of any observed differences in *T*_2_ relaxation times. Further we have investigated whether *T*_2_ mapping is capable of detecting changes in morphology or perfusion in two mouse models of perturbed pregnancy. The reduced uterine perfusion pressure (RUPP) model, where perfusion is deliberately reduced in order to mimic the oxidative and inflammatory stress in the first “stage” of preeclampsia [21]–[22], and the inflammatory cytokine imbalance (TNF-α infusion) model [23]–[24] were utilized in order to enable the examination of links between structural abnormalities in the placenta, altered perfusion, and the downstream effects that lead to the maternal hypertensive response.

## Methods

### Ethics Statement

All procedures were approved by the University of Western Sydney Animal Care and Ethics Committee (Animal Research Authority #A6668) and follow the “Guidelines to Promote the Wellbeing of Animals used for Scientific Purposes” as laid out by the National Health and Medical Research Council of Australia. Animals were monitored twice daily post surgical interventions and additional analgaesia was provided if required. Animals (dams and fetuses) were euthanized by cervical dislocation prior to tissue collection.

### Animals

C57BL/6JArc mice were obtained from the Animal Resource Centre (Canning Vale, WA, Aus) and were housed in a temperature controlled room in individually ventilated cages (up to 5 per cage), maintained in a 12:12-h light-dark cycle with *ad libitum* access to water and standard rodent chow. Animals were time-mated and on day 13.5 of gestation were randomly assigned to either RUPP (*n* = 3), TNF-α infusion (*n* = 3) or normal pregnant (*n* = 3), sham surgery (*n* = 2) or saline infusion (*n* = 2) control groups and housed individually until termination of the experiment. Animals were subject to MRI on day 17.5 of gestation unless otherwise indicated.

### Reduced utero-placental perfusion pressure (RUPP) procedure

The RUPP procedure has been described in both rats [21] and baboons [22] to induce placental ischaemia and hypertension. For the study undertaken here the procedure was altered to perform a unilateral ligation of the right uterine artery only as ligation of both branches of uterine artery and the lower abdominal aorta resulted in a high rate of abortion in the mice. Briefly, a silk suture was tied around the right uterine artery proximal to the ovarian artery on day 13.5 of gestation. Normal pregnant mice underwent a sham surgery on the same day of pregnancy. Surgical anaesthesia was induced with an intra-peritoneal injection of ketamine (100 mg/kg) and xylazine (10 mg/kg). Analgaesia was administered pre-operatively via subcutaneous injection (buprenorphine 0.1 mg/kg).

### TNF-α Infusion

TNF-α infusion has been utilized in both baboons [23] and rats [24] to induce hypertension in pregnancy. For this study TNF-α was infused via a subcutaneous implantation of a mini-osmotic pump (Model 1007D, Alzet, Cupertino, CA) designed to deliver a constant release of TNF-α (500 ng/kg/day). Briefly, an incision was made into the skin below the right scapulae on day 13.5 of gestation and the mini-osmotic pump primed with TNF-α was inserted into a subcutaneous pocket. Control animals received a saline implant. Anaesthesia and analgaesia was as described for RUPP surgery.

### Magnetic Resonance Imaging (MRI)

^1^H MRI images were taken of anaesthetised mice placed in a vertical animal probe using a Bruker Avance 11.74 Tesla wide-bore spectrometer with micro-imaging probe capable of generating gradients of 0.45 T/m. Anaesthesia was induced with 4% isofluorane in a chamber before the animals were transferred supine to the animal imaging probe. Isofluorane (lowered to 2%) was continuously delivered via a nose cone at an air flow of 150 ml/min. A small collar was used to maintain their head in a vertical position during scanning. A pressure sensitive pillow was taped to their abdomen to monitor respiration, the mice were wrapped for insulation and the animal chamber of the imaging probe was maintained at around 28 °C. The probe was inserted vertically into the scanner and the isofluorane concentration reduced to 1.5–1.7% and titrated to a respiration rate of approximately 50–60 breaths per minute. Sequence acquisition was gated on respiration (Model 1025 Small Animal Monitoring and Gating System, SA Instruments Inc, Stony Brook, NY, USA) in order to reduce motion artifacts. A Gradient Echo (GEFI) sequence protocol was used to obtain a series of localising images across the abdominal region. Thirty contiguous 1 mm slices, with an in plane resolution of 0.25 mm in either axial or coronal plane and high resolution images (in plane resolution of 0.12 mm) were taken of selected slices. *T*_2_ measurements using the same geometry were also acquired using a Multi Slice Multi Echo (MSME) sequence protocol (Bruker MSME-*T*_2_-map) with a 10 ms echo time and an in-plane resolution of 0.1–0.2 mm. MATLAB (The Mathworks, Natick, MA, USA) was used to generate *R*_2_ (1/*T*_2_) maps from the acquired data using non-linear least squares regression using the Levenberg-Marquardt-Fletcher algorithm. For quantification, *T*_2_ values were calculated from three points in each selected region of interest within 2–5 individual placentas. *T*_1_ maps were also produced for some placenta using MATLAB from data acquired using the TrueFISP sequence in FID mode with a flip angle of 5 degrees, 16 frames and a repetition time of 5 ms [25]. Additional *T*_2_ measurements were acquired on the same slices of one normal pregnant mouse immediately after blood flow was reduced to zero by terminal anaesthesia.

### Histology

Following MRI at gestational day 17.5, animals were euthanased, placentas collected, rinsed in PBS and fixed for 24 h in 10% formalin (Sigma) at 4 °C and processed into paraffin blocks prior to sectioning into 5 µm slices. Antigen retrieval was performed using proteinase K for 7 min (Dako Aust Campbellfield, Vic, Aus) and sections were immunostained for mouse cytokeratin (pAb Z0622; 1/1000, Dako Aust, Campbellfield, Vic, Aus), visualised with DAB (Envision kit, Dako Aust) and counterstained with haemotoxylin. Microscopy was performed with a Zeiss LSM 510 confocal microscope.

### Statistics

Statistical analysis was carried out using SPSS software (version 20) (SPSS, Inc., Chicago, IL, USA). Generalized linear modelling using linear regression was used to evaluate the differences in *T*_2_ values between regions in 5 placentas before and after blood flow. Generalized Estimating Equation Modelling clustering placenta within animals and animals within treatment groups was used to evaluate the differences in *T*_2_ values and the *T*_2_lab/*T*_2_junc ratios. Data are expressed as means ± SE with the level of significance being *p*<0.05. A logarithmic transformation was carried out on ratios prior to statistical analysis.

## Results

### Distinct regions of T_2_ contrast in murine placenta

**Figure 1:**
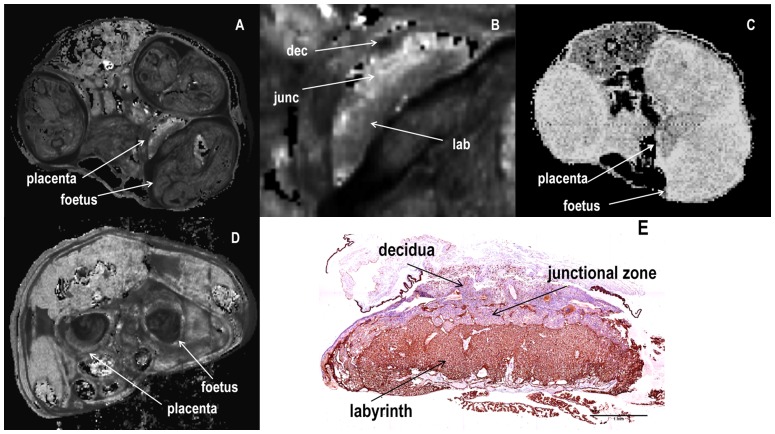
MRI images of normal gestational day 17.5 pregnant mouse. **(A)** *R*_2_ ( =  1/*T*_2_) map of abdominal cross section. **(B)** Enlarged placenta from (A) showing three distinct regions of labyrinth(lab), junctionasl zone (junc) and deciduas (dec); **(C)** *T*_1_ map of the same slice; **(D)** *R*_2_ ( =  1/*T*_2_) map at gestational day 10.5. **(E)** Histological section immunostained for cytokeratin showing position of trophoblast cells (brown) in the labyrinth.

Three distinct morphological regions of contrast based on *T*_2_ relaxation times were discerned in the MRI images of normal pregnant mouse placenta at gestational day 17.5 correlating to the labyrinth, junctional and decidual zones (Figure 1A and B). No variation of contrast within areas of the placenta was observed in the *T*_1_ images (Figure 1C). *T*_2_ mapping did not differentiate placental regions at gestational day 10.5 prior to the establishment of maternal circulation in the mouse placenta [26] (Figure 1D).

### T_2_ contrast abolished at loss of blood flow

**Figure 2:**
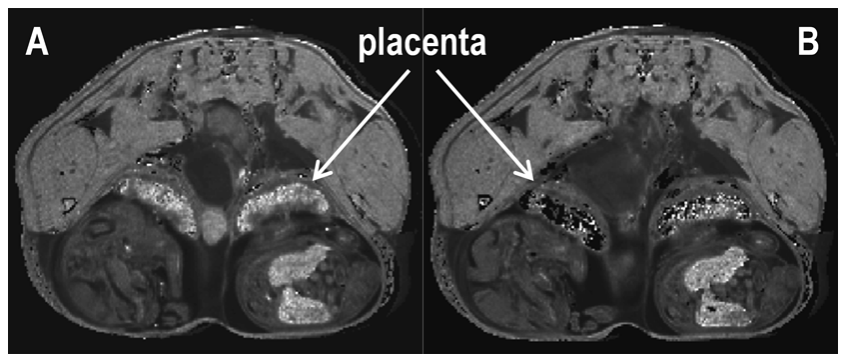
*T*_2_ contrast in the placenta is abolished at loss of blood flow. **(A)** *R*_2_ (1/*T*_2_) map of gestational day 17.5 normal pregnant C57BL/6JArc mouse showing *T*_2_ contrast in the different regions of the placenta. **(B)** *R*_2_ (1/*T*_2_) map of same abdominal slice after blood flow had ceased.

**Figure 3:**
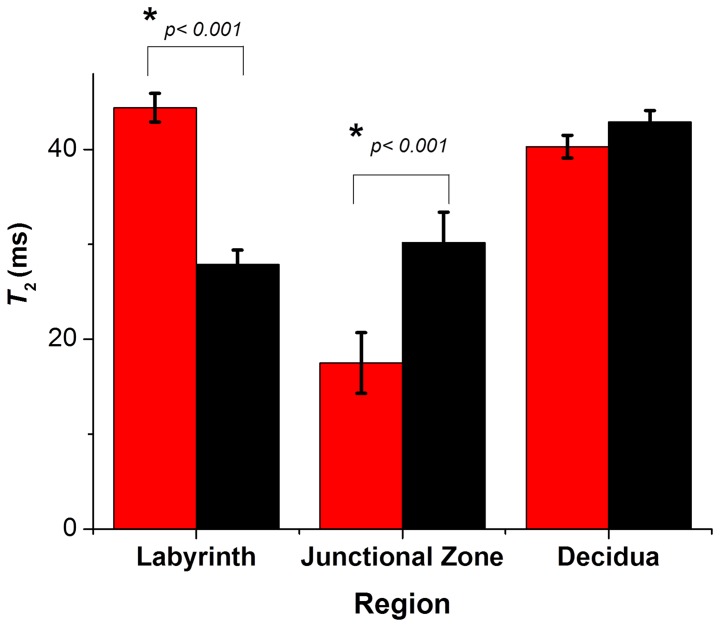
Comparison of *T*_2_ values from different regions of the placenta at day 17 of gestation during blood flow (red) and after blood flow has ceased (black). *T*_2_ values were calculated from 3 points in each region from the same 5 placentas before and after blood flow ceased.

To determine the contribution of perfusion to the contrast between regions in the *R*_2_ (1/*T*_2_) map, additional *T*_2_ measurements were acquired on the same slices of one normal pregnant mouse immediately after the blood flow was reduced to zero by terminal anaesthesia. Upon cessation of blood flow the difference in *T*_2_ contrast between the three regions was substantially reduced (Figure 2). There was a significant decrease in *T*_2_ contrast in the labyrinth (*p*<0.001) and a significant increase in the junctional region (*p* = 0.003) upon loss of blood flow, whereas the *T*_2_ values of the decidual region remained unchanged (*p* = 0.21) (Figure 3). The ratio of *T*_2_lab/*T*_2_junc was calculated to further clarify the observed changes, decreasing from 2.56 ±0.14 during blood flow to 1.04±0.14 after blood flow ceased (*p*<0.001).

### Pattern of T_2_ contrast altered in perturbed pregnancies

**Figure 4:**
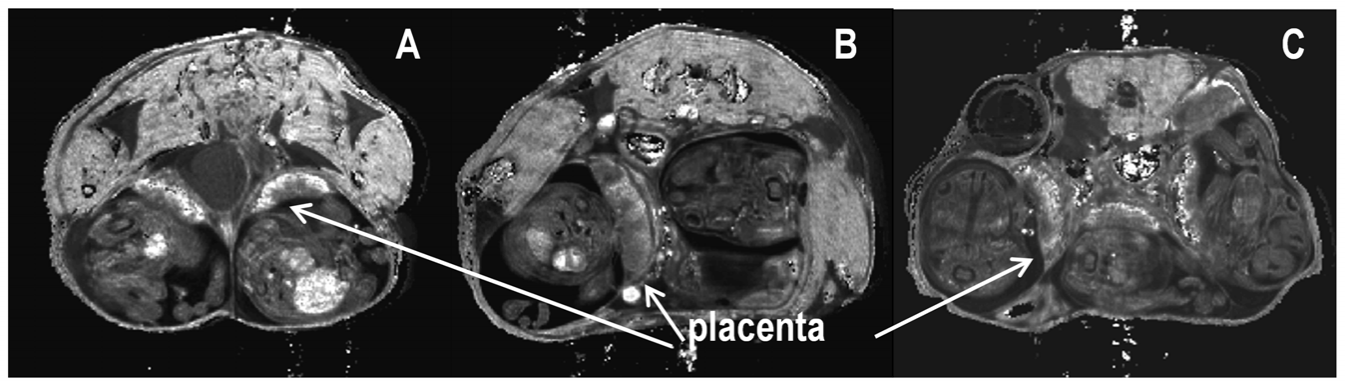
The pattern of *T*_2_ contrast is altered in perturbed pregnancies. **(A)** *R*_2_ (1/*T*_2_) map of gestational day 17.5 pregnant C57BL/6JArc mouse showing contrast in the placenta of normal pregnant animal; **(B)** RUPP animal and **(C)** TNF-α treated mouse.

**Figure 5:**
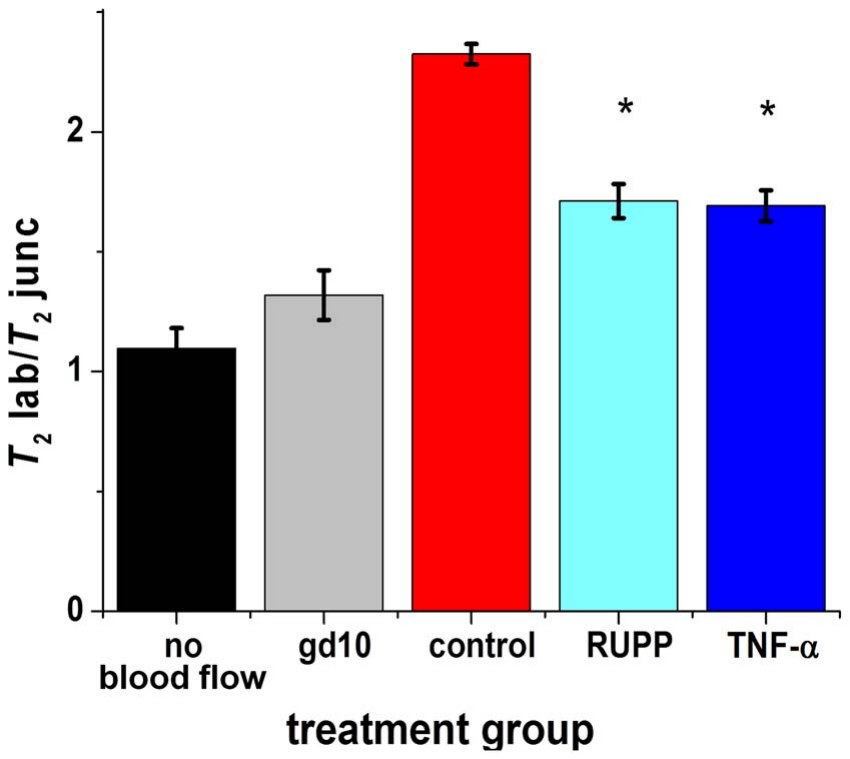
Comparison of the ratio of *T*_2_lab/ *T*_2_junc values between treatment groups. *T*_2_ values were measured at gestational day 17 of control (red) and perturbed pregnancy model animals (light and dark blue) and at loss of blood flow (black). *T*_2_ values were also measured at gestational day 10, prior to establishment of maternal placental blood flow (grey). The ratio of *T*_2_lab/ *T*_2_junc were; no blood flow (*n* = 1)1.09 ± 0.09; gestational d10 (*n* = 1)1.32 ± 0.10; control (*n* = 8) 2.35 ± 0.06; RUPP (*n* = 3) 1.70 ± 0.16 (*p* = 0.001); TNF-α (*n* = 3) 1.74 ± 0.19 (*p* = 0.001). *T*_2_ values were calculated from 3 points in each region, from 2-5 placentas from each animal.

**Figure 6:**
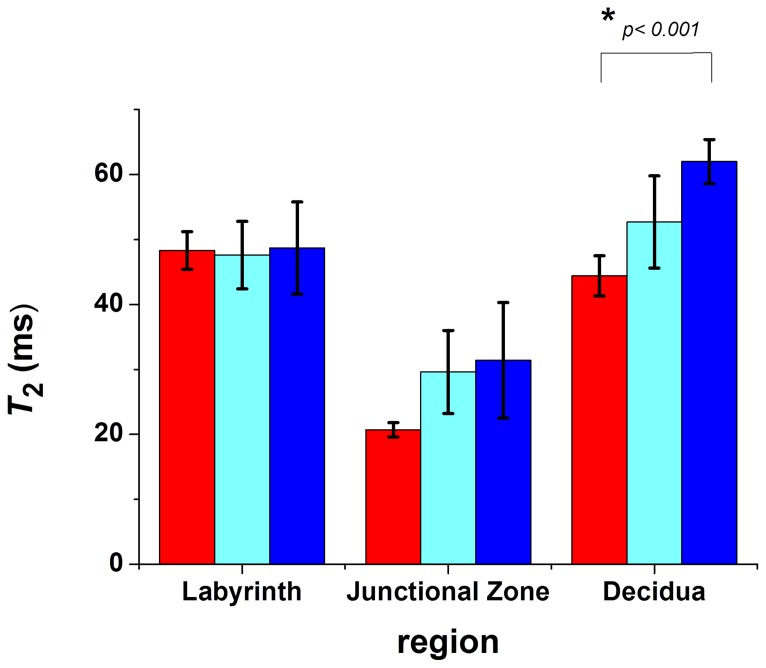
Comparison of the *T*_2_ values from different regions of the placenta. Control animals (*n* = 8) are shown in red; RUPP animals (*n* = 3) in light blue and TNF-α treated animals (*n* = 3) in dark blue. *T*_2_ values were calculated from 3 points in each of the labyrinth, junctional zone and decidua regions, from 2–5 placentas from each animal at day 17.5 of gestation.

We examined whether morphological differences could be detected by *T*_2_ mapping in the placenta of mice subjected to two experimental models of preeclampsia; namely the RUPP model and the inflammatory cytokine imbalance model (TNF-α). Differences in the pattern of the regions of *T*_2_ contrast in the placenta were observed between control, RUPP, and TNF-α treated mice (Figure 4). The ratio of *T*_2_lab/ *T*_2_junc was significantly altered in RUPP 1.70 ± 0.16 (*n* = 3, *p* = 0.001) and TNF-α treated, 1.74 ± 0.19 (*n* = 3, *p* = 0.001) animals compared to control animals 2.35 ± 0.06 (*n* = 8) (Figure 5), and there was a trend for larger *T*_2_ values in the junctional zone and deciduas of RUPP and TNF-α treated animals (Figure 6). Sham-operated and saline-infused controls were not significantly different to normal pregnant animals and for the purposes of analysis the three control groups were grouped together.

## Discussion

This study has shown that higher resolution *T*_2_ maps of mouse placenta can clearly differentiate between different regions of the placenta at time points after the maternal circulation is fully established. This differs from previous studies of *T*_2_ relaxation times in humans using much lower field strengths, where the placenta was observed to be homogeneous [18], [27]. Our findings show that the labyrinth has a *T*_2_ relaxation time twice that of the junctional zone, with the observed contrast between these regions of the placenta being abolished on loss of blood flow. The decreased *T*_2_lab/*T*_2_junc ratio on loss of blood flow was due to both a decrease in the *T*_2_ value in the labyrinth and an increase in the *T*_2_ value in the junctional zone.

Examining both the structure of the placenta and the influences on *T*_2_ relaxation times provides an explanation of the differences between regions and of the changes on loss of blood flow. In the labyrinth, essentially an intermeshed network of independent fetal and maternal blood vessels, there is an abundance of both freely moving protons, in the form of free water, and of blood cells containing mostly highly oxygenated haemoglobin. The junctional zone is dense with spongiotrophoblast and giant trophoblast cells. The longer *T*_2_ value measured in the labyrinth, compared to the more cellularly dense junctional zone, can be accounted for by the abundance of freely moving protons in this region which give rise to longer T_2_ relaxation times [13]. Upon cessation of blood flow, the *T*_2_ value in the labyrinth decreased and the *T*_2_ value in the junctional zone increased. After cessation of blood flow the tissue continues to metabolise for some time, consuming O_2_, producing CO_2_ and generating deoxyhaemoglobin. The decrease in the *T*_2_ value in the labyrinth can be accounted for by an increase in the paramagnetic ion deoxyhaemoglobin which gives rise to shorter *T*_2_ relaxation times [14]–[15], however in the junctional zone there are few haemoglobin containing blood cells and hence minimal paramagnetic effect of deoxyhaemoglobin on the *T*_2_ value in this region. Conversely, due to the build up of CO_2_ in the tissue and the consequent acidosis, the increase in free protons would account for the observed increase in the *T*_2_ value in the junctional zone. The effects of pH on *T*_2_ values has been well documented in both muscle tissue of live patients [16] and in isolated muscle [17], with a clear correlation between decrease of intracellular pH and an increase in the *T*_2_ relaxation time. While acidosis would also occur in the labyrinth the predominant effect on the *T*_2_ value in this region appears to be that of the paramagnetic deoxyhaemoglobin. Thus, the abolition of contrast between regions of the placenta upon complete loss of blood flow is consistent with the effects on *T*_2_ relaxation times by both increases in deoxyhaemoglobin (hypoxia) and decreases in intracellular pH (acidosis).

The RUPP and the TNF-α treated mice also showed a decrease in contrast between the labyrinth and junctional zones, though this is primarily due to a trend for an increase in the *T*_2_ value in the junctional zone alone with no change in the labyrinth observed. While the observed changes within each region have been limited to trends by the small number of mice in this study, the large number of individual placenta examined within each litter and the significance of the individual placental *T*_2_lab/*T*_2_junc ratio suggests that the trends are real. In the RUPP mice where blood flow is being artificially constricted, lower perfusion pressures are assumed to lead to hypoxia, and presumably, a greater proportion of deoxyhaemoglobin. In turn, this should lead to a lower *T*_2_ value in the labyrinth, however this is not the case and the observed decrease in the *T*_2_lab/*T*_2_junc ratio is due solely to an increase in the *T*_2_ value in the junctional zone. It could be speculated that, although reduced, the blood flow may be sufficient to prevent an accumulation of deoxyhaemoglobin in the labyrinth resulting in no observed change in the *T*_2_ value. Alternatively, there may be a small increase in deoxyhaemoglobin tending to reduce *T*_2_, however it is counteracted by a simultaneous increase in pH tending to increase *T*_2_, thereby providing no net change. Conversely in the junctional zone, the observed increase in the *T*_2_ value is likely to be a reflection of an increased acidosis in this region. The fact that an increase in inflammatory cytokine (TNF-α) shows a similar *T*_2_ profile to RUPP suggests that cellular acidosis may be a common pathway in these two models, implicating intracellular acidosis in the regulation of signalling pathways leading to the downstream effects on the maternal system. Further studies involving the measurement of intracellular pH using ^31^P MRI would be warranted to confirm the tissue acidosis in these models.

Structural abnormalities in the placenta have been proposed to result in reduced placental perfusion [28] or ischaemia-reperfusion injury [29], and to lead to placental oxidative stress, cellular damage and inflammation. The subsequent release of anti-angiogenic and other toxic compounds into the maternal circulation has been shown to lead to endothelial dysfunction and the maternal hypertensive response in the preeclamptic pregnancy [30]. The signalling pathways leading to the release of the anti-angiogenic molecules are as yet unclear, but may involve both oxygen dependent elements such as hypoxia inducible factor-1 (HIF-1) [31] or Jumonji domain-containing protein 6 (Jmjd6) [32], and non oxygen dependent mechanisms such as toll-like receptor-3 (TLR-3) and NFKB pathways [33]. Our data suggests that pH dependent mechanisms may also play a significant role, and may be as equally important as hypoxia in the perturbed placenta. Recently it has been reported that coupling factor 6 (CF6) activation of ectopic ATP synthase leads to increased sFLT-1 through intracellular acidosis induced c-Src signalling [34]. CF6 is found in the circulation, has higher levels in spontaneously hypertensive rats and its release from the surface of endothelial cells is stimulated by TNF-α [34]. Many other studies have shown that acidosis and hypoxia have a linked role in some signalling pathways [35]–[38] and our data would be in keeping with these findings.

In conclusion, this study has shown that high resolution *T*_2_ maps of mouse placenta can distinguish different regions of the placenta and that, upon loss of blood flow, contrast between regions is abolished consistent with effects on *T_2_* relaxation times by both increases in deoxyhaemoglobin (hypoxia) and decreases in intracellular pH (acidosis). We have demonstrated a potential for this technology to correlate dynamic perfusion related changes with structural alterations and imbalances in inflammatory cytokines and anti-angiogenic molecules in experimental models of perturbed pregnancies. As a result of our findings we postulate that acidosis as well as hypoxia may be a feature of pregnancy complications and may play a role in signalling pathways that lead to downstream effects.
